# Exosomal miR-223 derived from natural killer cells inhibits hepatic stellate cell activation by suppressing autophagy

**DOI:** 10.1186/s10020-020-00207-w

**Published:** 2020-09-01

**Authors:** Ling Wang, Yinghao Wang, Jun Quan

**Affiliations:** 1 Department of Infectious Diseases, Xiangya Hospital of Central South University, No. 87 Xiangya Road, Changsha, 410008 Hunan China; 2 Department of Ophthalmology, The Second Xiangya Hospital of Central South University, Changsha, 410011 Hunan China

**Keywords:** Hepatic stellate cell activation, Natural killer cell, Exosome, miR-223, Autophagy

## Abstract

**Background:**

Activation of hepatic stellate cells (HSCs) is a prominent driver of liver fibrosis. We previously demonstrated that exosomes derived from natural killer (NK) cells (NK-Exo) attenuated TGF-β1-induced HSC activation. Herein, this study was designed to investigate the mechanism underlying the action of NK-Exo.

**Methods:**

NK-Exo was isolated from NK-92MI cells and then administered into TGF-β1-treated LX-2 (human HSC line) cells. MiR-223 expression in NK-Exo was downregulated by transfecting NK-92MI cells with miR-223 inhibitor followed by exosome isolation. The HSC activation was evaluated by determining cell proliferation using CCK-8 assay and measuring the protein levels of α-SMA and CoL1A1 using western blot in LX-2 cells. The expression of miR-223 was detected by qRT-PCR. The interaction between miR-223 and ATG7 was analyzed by a dual-luciferase activity assay. The autophagy was evaluated by measuring the autophagy-related proteins using western blot.

**Results:**

miR-223 was highly expressed in NK-Exo and inhibition of miR-223 expression in NK-Exo abrogated the inhibitory effect of NK-Exo on TGF-β-induced HSC activation. ATG7 was confirmed as a direct target of miR-223. Furthermore, treatment with the autophagy activator rapamycin and ATG7 overexpression in LX-2 cells abolished the HSC activation-suppressive effect of NK-Exo.

**Conclusion:**

NK-Exo attenuated TGF-β-induced HSC activation by transferring miR-223 that inhibited autophagy via targeting ATG7.

## Background

Activation of hepatic stellate cells (HSCs) is a prominent driver of liver fibrosis that can eventually lead to cirrhosis, liver failure, and even liver cancer (Higashi et al. 2017; Parola and Pinzani 2019). As such, effective therapeutic strategies for inhibiting HSC activation are urgently needed to reverse liver fibrosis. Exosomes are nano-sized membrane vesicles (30–150 nm in diameter) that can be released from various cell types (Chen et al. 2020). Natural killer (NK) cells are important effector cells in many innate immune processes and play an important regulatory role in HSC activation (Fasbender et al. 2016; Foley et al. 2011). NK cells can influence the biological functions of recipient cells through secretion of exosomes (Shoae-Hassani et al. 2017; Neviani et al. 2019). Our group has previously demonstrated that exosomes derived from NK cells (NK-Exo) inhibited TGF-β1-induced HSC activation in HSC-LX-2 cells and carbon tetrachloride (CCl_4_)-induced liver fibrosis in BALB/c mice (Wang et al. 2020a). However, the underlying mechanism of NK-Exo action remains unclear.

As a crucial means of intercellular communication, exosomes can transfer specific cargos including microRNAs (miRNAs) from the originating cells to the recipient cells (Zhang et al. 2019; Su et al. 2019). miRNAs are endogenous, small (19–22 nucleotides) RNA molecules that regulate gene expression through post-transcriptional pattern by directly binding to the 3′-untranslated region (3′-UTR) of target mRNAs (Liu et al. 2018). Emerging evidence has indicated that miRNAs play a regulatory role in the occurrence and progression of liver fibrosis by influencing HSC activation (Zhao et al. 2019). miR-223 plays an essential role in the pathogenesis of various types of liver diseases, such as hepatitis virus infections, alcohol- or drug- induced liver injury, cirrhosis, and liver cancer (Ye et al. 2018). A recent study showed that treatment with miR-223-3p significantly mitigated fibrosis development and HSC activation in a murine model of fibrotic nonalcoholic steatohepatitis (NASH) (Jimenez Calvente et al. 2020).

Autophagy is a homeostatic, catabolic degradation process that degrades damaged cellular proteins and organelles to maintain cellular metabolism. Autophagy is induced during HSC activation and blockage of autophagy inhibits liver fibrosis by inhibiting HSC activation (Ye et al. 2020). Thus, autophagy may represent a potential target for developing anti-fibrotic strategies.

Autophagy-related 7 (ATG7), an autophagy marker, was identified as a putative target of miR-223 using Targetscan analysis (http://www.targetscan.org). Recently, Neviani et al. isolated NK cells from peripheral blood of healthy donors and profiled the top miRNAs represented in the exosomes. They found that miR-223 was highly expressed in NK cell-derived exosomes (Neviani et al. 2019). Thus, we hypothesized that NK cells might transfer miR-223 via exosomes to HSC-LX-2 cells where miR-223 suppressed autophagy via targeted inhibition of ATG7 expression, thereby attenuating TGF-β1-induced HSC activation.

## Materials and methods

### Cell culture

The human NK cell line (NK92-MI; ATCC, Manassas, VA, USA) was cultured maintained in stem cell growth medium (Cellgro, Freiburg, Germany) containing 2% exosome-depleted human serum and 1% penicillin-streptomycin. The human HSC line (LX-2; ATCC) was cultured in DMEM (Gibco, Grand Island, NY, USA) supplemented with 10% FBS (Gibco), glutamine, sodium pyruvate, and 1% penicillin-streptomycin. Cells were maintained in a humidified air with 5% CO_2_ at 37 °C.

### Cell transfection

The miR-223 mimic, miR-223 inhibitor, mimic negative control (NC), inhibitor NC, ATG7 overexpression vector (pcDNA3.1-ATG7), and empty vector were purchased from GenePharma (Shanghai, China). NK-92MI cells at 70–80% confluence were transfected with miR-223 inhibitor or inhibitor NC at a final concentration of 50 nM. LX-2 cells were transfected with these mimics, inhibitors, or vectors according to the experimental design. Cell transfection was performed using the Lipofectamine 3000 reagent (Invitrogen, Carlsbad, CA, USA). At 48 h post-transfection, cells were harvested for further assays.

### Isolation of exosomes from NK cells

NK-92MI cells were used for exosome isolation after cultivation for 3 days according to a previous study (Zhu et al. 2017). When the cell confluence reached 70%, the media were replaced with exosome-depleted FBS (Gibco). After incubation for 48 h, cell supernatant was collected and centrifuged at 1500×g for 3 min, 2000×g for 15 min, and then 3000×g for 20 min at 4 °C to remove dead cells and cell debris. The supernatant was then centrifuged at 100,000×g for 1 h to obtain exosomes. The precipitated exosome pellets were resuspended in PBS and stored at − 80 °C until further use. The exosomes derived from untreated NK-92MI cells, miR-223 inhibitor-transfected NK-92MI cells, or inhibitor NC-transfected NK-92MI cells were referred to as NK-Exo, NK-Exo-miR-223I, or NK-Exo-NC, respectively.

### Characterization of exosomes

The morphological characteristics of the exosomes were identified under transmission electron microscopy (TEM; Hitachi, Tokyo, Japan) as previously described (Yang et al. 2019). The nanoparticle tracking analysis was performed on a NanoSight NS500 (Malvern Instruments, Malvern, UK) to evaluate the size distribution of exosomes. Western blot was performed to detect the protein levels of exosomal surface markers (ALIX and CD63).

### Cell treatment

LX-2 cells were treated with TGF-β1 (5 ng/mL) for 24 h to stimulate HSC activation. LX-2 cells in the NK-Exo treatment groups were pretreated with NK-Exo (10 μg/mL) before TGF-β1 treatment. LX-2 cells in the rapamycin treatment groups were pretreated with the autophagy activator rapamycin (2 mM) in DMSO for 12 h before TGF-β1 treatment.

### Luciferase reporter assay

The interaction between miR-223 and 3′-UTR of ATG7 was verified by the luciferase reporter assay. Briefly, LX-2 cells at 80% confluence were co-transfected with miR-223 mimic/mimic NC, ATG7 wild-type (WT)/ATG7 mutated (Mut) luciferase constructs, and the internal control pRL-TK (Promega, Madison, WI, USA) using Lipofectamine 2000 (Invitrogen). At 24 h post-transfection, the luciferase activity was determined using a luciferase reporter assay system (Promega).

### Cell proliferation assay

LX-2 cells were seeded into 96-well plates at a density of 5 × 10^3^ cells/well and were given different treatments. After 48 h, the CCK-8 reagent (Beyotime, Haimen, China) was added to each well and cells were incubated at 37 °C for 4 h. The OD_450_ values of different treatment groups were measured with a microplate detector (EnSpire 2300, PerkinElmer, Waltham, MA, USA).

### Quantitative real-time PCR (qRT-PCR)

After different treatments, the cells or exosomes were lysed and total RNA was extracted using TRIzol reagent (Invitrogen). After reverse transcription, the expression of miR-223 was detected using the miRNA qRT-PCR kit (GeneCopoeia, Rockville, MD, USA) in Applied Biosystems 7500 PCR system (Applied Biosystems, Foster, CA, USA). The mRNA levels of α-smooth muscle actin (α-SMA), collagen type I alpha 1 chain (CoL1A1) were detected using a LightCycler® 480 SYBR Green I Master qPCR mix (Roche, Mannheim, Germany). The specific primers were as follows: miR-223-Forward, 5′- CGTGTATTTGACAAGCTG − 3′; and miR-223-Reverse, 5′- GAACATGTCTGCGTATCTC -3′; U6-Forward, 5′ TGCGGGTGCTCGCTTCGCAGC- -3′; and U6-Reverse, 5′- CCAGTGCAGGGTCCGAGGT − 3′; α-SMA-Forward, 5′ - GACAGCTACGTGGGTGACGAA − 3′; and α-SMA-Reverse, 5′- TTTTCCATGTCGTCCCAGTTG − 3′; CoL1A1-Forward, 5′ - GATTCCCTGGACCTAAAGGTGC − 3′; and CoL1A1-Reverse, 5′- AGCCTCTCCATCTTTGCCAGCA -3′; GAPDH-Forward, 5′- GTCTCCTCTGACTTCAACAGCG − 3′; and GAPDH-Reverse, 5′- ACCACCCTGTTGCTGTAGCCAA − 3′; Expression fold changes were calculated using the 2^-ΔΔCt^ method. U6 was used as the reference gene for miR-223. GAPDH was used as the reference gene for α-SMA and CoL1A1.

### Western blot

Western blot analysis was performed as previously described (Wang et al. 2020b). After treatment, the cells or exosomes were lysed and total protein was extracted using the radioimmunoprecipitation assay lysis buffer (Beyotime). Proteins were electrophoresed by sodium dodecyl sulfate-polyacrylamide gel electrophoresis gels and transferred to polyvinylidene fluoride membranes (Millipore, Billerica, MA, USA). Then membranes were incubated overnight with primary antibodies against ALIX (1:1000, Abcam, Cambridge, MA, USA), CD63 (1:1000; Abcam), α-SMA (1:1000; Abcam), CoL1A1 (1:1000; Abcam), p62 (1:1000; Santa Cruz Biotechnology, Dallas, TX, USA), Beclin-1 (1:1000, Santa Cruz Biotechnology), LC3 (1:1000; Santa Cruz Biotechnology), ATG7 (1:1000; Abcam), and GAPDH (1:3000; Abcam). The membranes were then incubated with the horseradish peroxidase-conjugated secondary antibodies (1:2000; Santa Cruz Biotechnology) for 1 h at room temperature.

### Statistical analysis

SPSS 22.0 (IBM, Chicago, IL, USA) was used to perform statistical analyses. The student’s *t*-test was used for comparison of two groups and ANOVA was used for multiple comparisons. *P* < 0.05 was considered statistically significant.

## Results

### Characterization of exosomes

Exosomes were isolated from the conditioned medium derived from NK-92MI cells, namely NK-Exo. TEM micrographs showed that the isolated exosomes were spherical with a size distribution between 0 ~ 150 nm in diameter (Supplementary Fig. 1A and B). Furthermore, western blot analysis showed that the isolated exosomes positively expressed exosomal markers including ALIX and CD63 **(**Supplementary Fig. 1C**)**.

### NK-Exo-mediated transfer of miR-223 attenuated TGF-β1-induced HSC activation

**Fig. 1:**
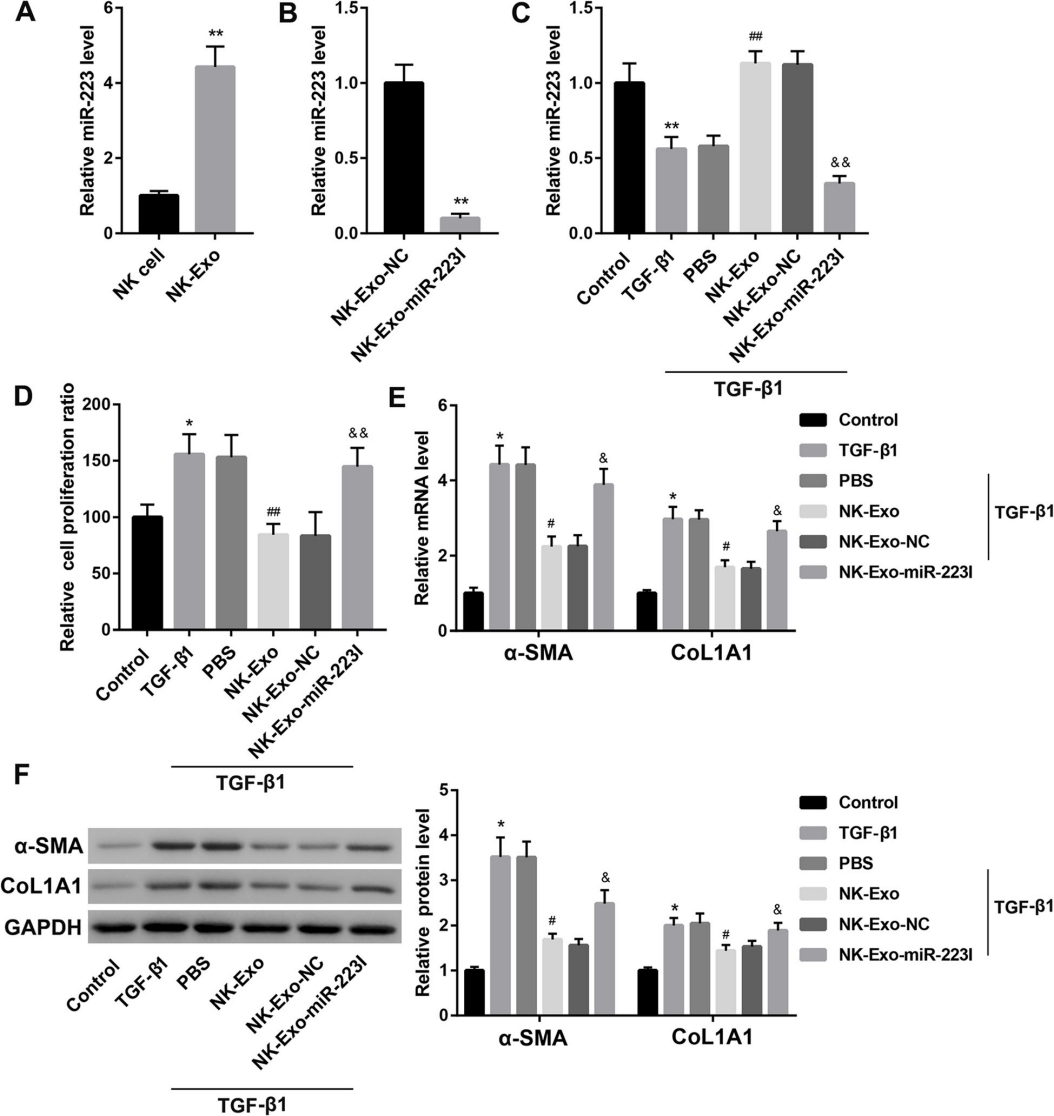
NK-Exo attenuated TGF-β1-induced HSC activation by transferring miR-223. **a** The relative expression of miR-223 determined by qRT-PCR in NK-92MI cells and NK-92MI cell-derived exosomes (NK-Exo). ^**^P < 0.01 vs. NK cell. **b** The relative expression of miR-223 determined by qRT-PCR in exosomes derived from NK-92MI cells transfected with inhibitor NC (NK-Exo-NC) or miR-223 inhibitor (NK-Exo-miR-223I). ^**^P < 0.01 vs. NK-Exo-NC. The relative expression of miR-223 determined by qRT-PCR **(c)**, cell proliferation ratio determined by CCK-8 assay **(d)**, the mRNA **(e)** and protein **(f)** levels of α-SMA and CoL1A1 in LX-2 cells in the groups of Control, TGF-β1, TGF-β1 + PBS, TGF-β1 + NK-Exo, TGF-β1 + NK-Exo-NC, and TGF-β1 + NK-Exo-miR-223I. ^*^P < 0.05, ^**^P < 0.01 vs. Control; ^#^*P* < 0.05, ^##^*P* < 0.01 vs. TGF-β1 + PBS; ^&^*P* < 0.05, ^&&^*P* < 0.01 vs. TGF-β1 + NK-Exo-NC. The data are presented as the mean ± standard deviation (*n* = 3)

Results of the qRT-PCR analysis showed that miR-223 expression was markedly higher in NK-Exo than that in parental NK-92MI cells **(**Fig. 1a**)**. To assess whether NK-Exo regulates HSC activation by transferring miR-223, we developed miR-223-deficient NK-Exo by transfecting NK-92MI cells with miR-223 inhibitor followed by exosomes isolation, namely, NK-Exo-miR-223I. miR-223 expression was confirmed to be sharply downregulated in NK-Exo-miR-223I when compared with the NK-Exo-NC group **(**Fig. 1b). TGF-β1 is important for HSC activation which is characterized by enhanced HSC cell proliferation and overproduction of CoL1A1 and α-SMA (Zheng et al. 2016). NK-Exo treatment rescued the TGF-β1-mediated downregulation of miR-223 expression in LX-2 cells **(**Fig. 1c). Of note, NK-Exo treatment significantly reduced the TGF-β1-induced elevation of cell proliferation ratio **(**Fig. 1d**)** and upregulation of mRNA and protein levels of α-SMA and CoL1A1 **(**Fig. 1e-f**)** in LX-2 cells. However, these effects of NK-Exo were counteracted when miR-223 expression in NK-Exo was inhibited **(**Fig. 1c-f**)**. These results suggested that NK-Exo attenuated TGF-β1-induced HSC activation by transferring miR-223.

### NK-Exo-mediated transfer of miR-223 attenuated TGF-β1-induced HSC activation by inhibiting autophagy

**Fig. 2:**
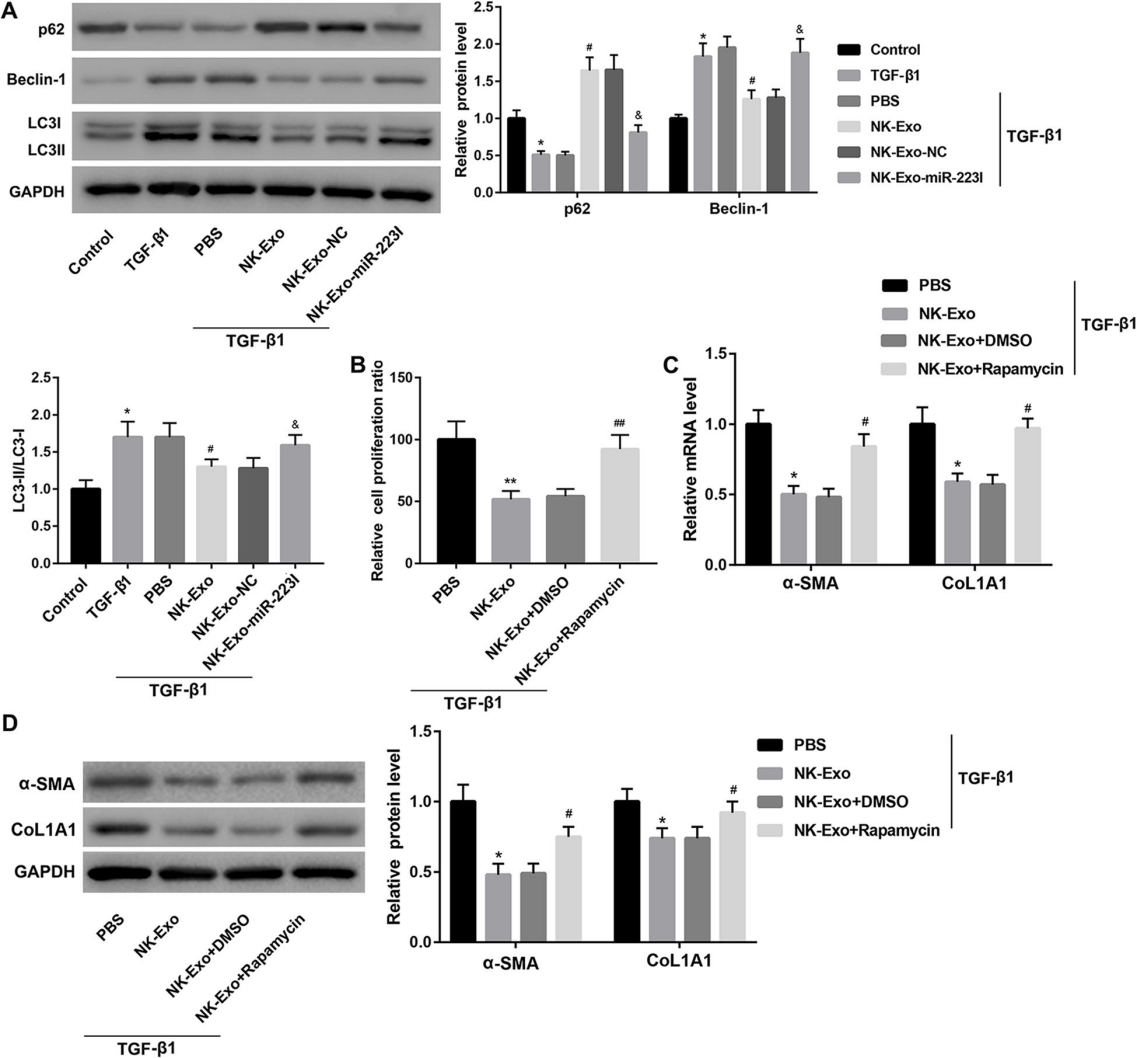
NK-Exo-mediated transfer of miR-223 attenuated TGF-β1-induced HSC activation by inhibiting autophagy. **a** The protein levels of autophagy-related proteins including p62, Beclin-1, LC3-I, LC3-II in LX-2 cells in the groups of Control, TGF-β1, TGF-β1 + PBS, TGF-β1 + NK-Exo, TGF-β1 + NK-Exo-NC, and TGF-β1 + NK-Exo-miR-223I. ^*^P < 0.05, vs. Control; ^#^*P* < 0.05, vs. TGF-β1 + PBS; ^&^*P* < 0.05, vs. TGF-β1 + NK-Exo-NC. Cell proliferation ratio determined by CCK-8 assay **(b)**, the mRNA **(c)** and protein **(d)** levels of α-SMA and CoL1A1 in LX-2 cells in the groups of TGF-β1 + PBS, TGF-β1 + NK-Exo, TGF-β1 + NK-Exo + DMSO and TGF-β1 + NK-Exo + rapamycin. ^*^P < 0.05, ^**^P < 0.01 vs. TGF-β1 + PBS; ^#^*P* < 0.05, ^##^*P* < 0.01 vs. TGF-β1 + NK-Exo + DMSO. The data are presented as the mean ± standard deviation (n = 3)

To determine whether autophagy is involved in exosomal miR-223-mediated attenuation of TGF-β1-induced HSC activation, we examined several autophagy-related proteins using western blot in LX-2 cells treated as described above. NK-Exo treatment significantly increased p62 protein level, whereas decreased Beclin-1 protein level and LC3-II/LC3-I ratio in TGF-β1-treated LX-2 cells. However, the autophagy-inhibitory effect of NK-Exo was compromised when miR-223 expression in NK-Exo was inhibited, suggesting that NK-Exo attenuated TGF-β1-induced autophagy by transferring miR-223 **(**Fig. 2a**)**.

To further verify whether NK-Exo attenuates TGF-β1-induced HSC activation by inhibiting autophagy, we treated LX-2 cells with PBS or NK-Exo in the absence or presence of rapamycin (an autophagy activator) under TGF-β1 stimulation. The cell proliferation ratio and the expression of α-SMA and CoL1A1 were significantly upregulated in the TGF-β1 + NK-Exo + rapamycin group when compared with the TGF-β1 + NK-Exo + DMSO group **(**Fig. 2b-d**)**. Collectively, these data indicated that NK-Exo-mediated transfer of miR-223 attenuated TGF-β1-induced HSC activation by inhibiting autophagy.

### NK-Exo-mediated transfer of miR-223 inhibited autophagy by targeting ATG7

**Fig. 3:**
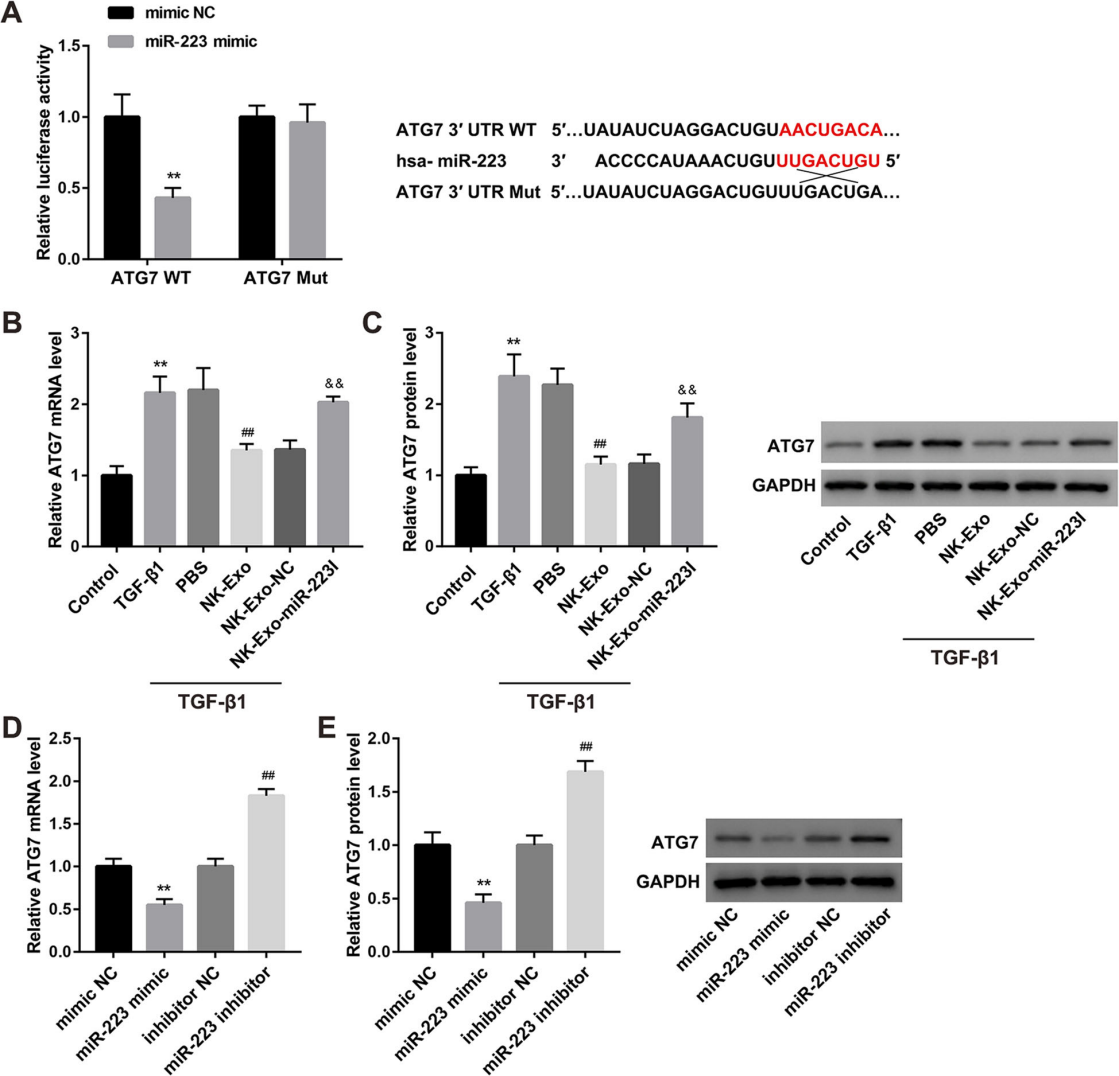
NK-Exo-mediated transfer of miR-223 inhibited autophagy by targeting ATG7. **a** The direct binding of miR-223 to ATG7 3′-UTR was verified by dual-luciferase reporter assay. ^**^P < 0.01 vs. mimic NC. The mRNA **(b)** and protein **(c)** levels of ATG7 in LX-2 cells in the groups of Control, TGF-β1, TGF-β1 + PBS, TGF-β1 + NK-Exo, TGF-β1 + NK-Exo-NC, and TGF-β1 + NK-Exo-miR-223I. ^**^P < 0.01 vs. Control; ^##^*P* < 0.01 vs. TGF-β1 + PBS; ^&&^*P* < 0.01 vs. TGF-β1 + NK-Exo-NC. The mRNA **(d)** and protein **(e)** levels of ATG7 in LX-2 cells transfected with mimic NC, miR-223 mimic, inhibitor NC, and miR-223 inhibitor. ^**^P < 0.01 vs. mimic NC; ^##^*P* < 0.01 vs. inhibitor NC. The data are presented as the mean ± standard deviation (n = 3)

To explore the molecular mechanism by which exosomal miR-223 inhibits autophagy, we performed luciferase activity reporter assay to verify whether ATG7 is a downstream target of miR-223. Data revealed that miR-223 mimic transfection significantly inhibited the luciferase activity of cells transfected with the ATG7 WT luciferase construct. Mutation of the binding sites could abolish the suppressing effect **(**Fig. 3a**)**, verifying that miR-223 directly targeted ATG7.

NK-Exo treatment diminished the TGF-β1-induced upregulation of ATG7 mRNA and protein levels in LX-2 cells. Inhibition of miR-223 expression in NK-Exo negated the suppressive effect of NK-Exo on ATG7 expression, indicating that NK-Exo inhibited ATG7 expression by transferring miR-223 **(**Fig. 3b-c**)**. To confirm the negative regulation of ATG7 expression in LX-2 cells by miR-223, we transfected LX-2 cells with miR-223 mimic and miR-223 inhibitor. The mRNA and protein levels of ATG7 were notably decreased following transfection with miR-223 mimic, whereas increased following transfection with miR-223 inhibitor **(**Fig. 3d-e**)**.

### NK-Exo attenuated TGF-β1-induced HSC activation by inhibiting ATG7 expression

**Fig. 4:**
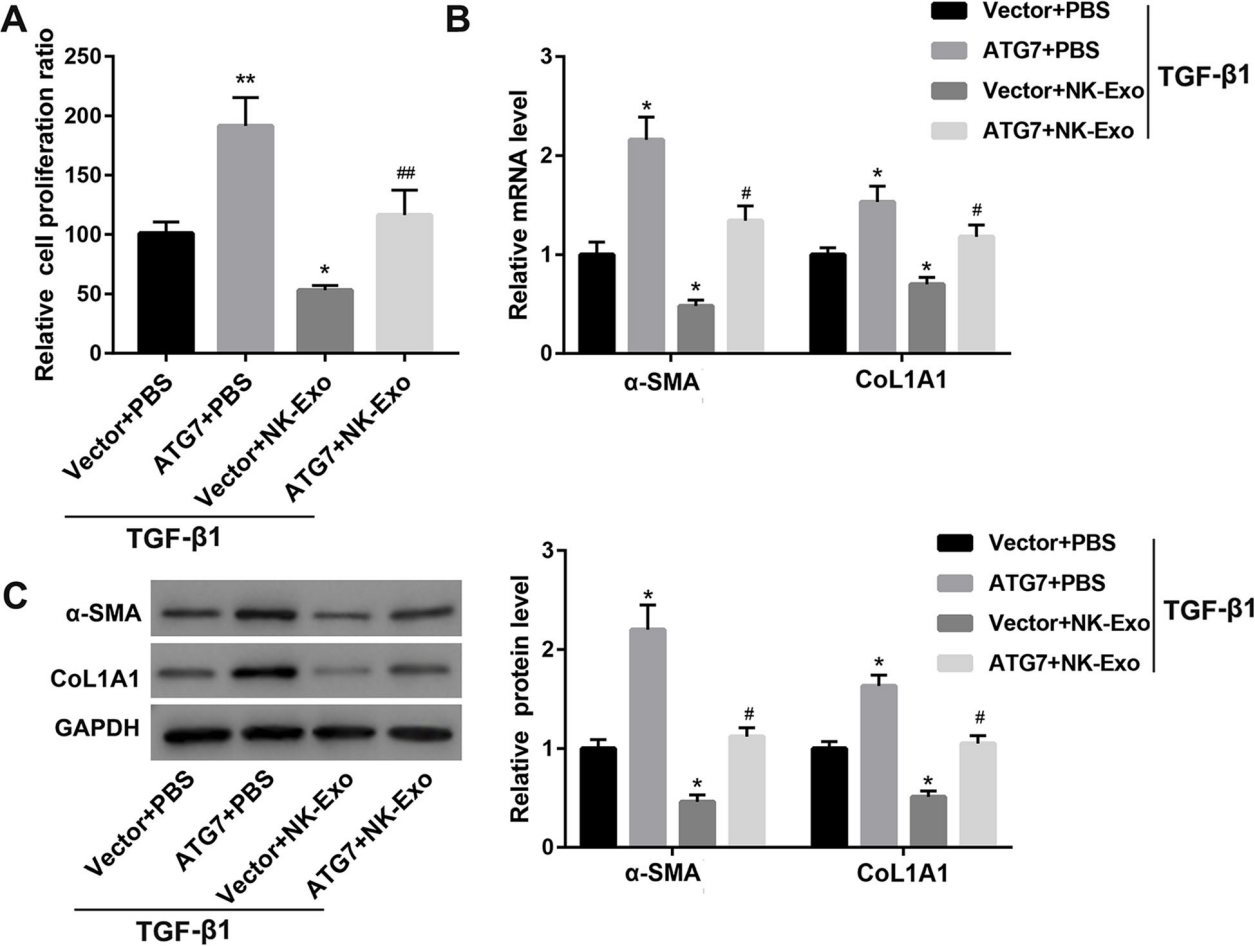
NK-Exo attenuated TGF-β1-induced HSC activation by inhibiting ATG7 expression. Cell proliferation ratio determined by CCK-8 assay **(a)**, the mRNA **(b)** and protein **(c)** levels of α-SMA and CoL1A1 in LX-2 cells transfected with ATG7 overexpression vector/empty vector followed by treatment with NK-Exo/PBS in the presence of TGF-β1. ^*^P < 0.05, ^**^P < 0.01 vs. TGF-β1 + Vector+PBS; ^#^*P* < 0.05, ^##^*P* < 0.01 vs. TGF-β1 + Vector+NK-Exo. The data are presented as the mean ± standard deviation (n = 3)

To examine the contribution of ATG7 to the suppressive effect of NK-Exo on HSC activation, we cultured ATG7-overexpressing LX-2 cells with NK-Exo in the presence of TGF-β1. Contrary to NK-Exo, ATG7 overexpression resulted in significant increases in cell proliferation ratio **(**Fig. 4a**)** and expression of α-SMA and CoL1A1 **(**Fig. 4b-c**)** in LX-2 cells. Importantly, the inhibitory effects of NK-Exo on cell proliferation ratio **(**Fig. 4a**)** and expression of α-SMA and CoL1A1 **(**Fig. 4b-c**)** were abolished when ATG7 expression was overexpressed in LX-2 cells.

## Discussion

HSCs are a prominent driver of liver fibrosis as the activated HSCs promote collagen deposition in the extracellular matrix by producing profibrogenic genes (α-SMA and CoL1A1), both of which are considered as markers of HSC activation (Yang et al. 2020). Thus it is essential to elucidate the mechanisms controlling HSC activation. TGF-β1 is a canonical HSC activator after liver injury (Prestigiacomo et al. 2020). In the present study, we found that miR-223 was highly expressed in NK-Exo and inhibition of miR-223 expression in NK-Exo significantly abrogated the TGF-β1-induced HSC activation, as demonstrated by suppressed proliferation and decreased expression of α-SMA and CoL1A1 in human HSC-LX-2 cells. NK cell-derived exosomes can influence the biological functions of recipient cells by transferring miRNAs (Neviani et al. 2019). Our results showed that NK-Exo attenuated TGF-β1-induced HSC activation by transferring miR-223.

Convincing evidence suggests that miRNAs are involved in regulating HSC activation and liver fibrosis. Zhou et al. demonstrated that miR-185 was lowly expressed in plasma of patients with hepatitis B virus-related cirrhosis, livers of mice with CCl_4_-induced liver fibrosis, and TGF-β1-activated HSC-LX-2 cells. They also found that miR-185 overexpression inhibited HSC activation and hepatic fibrosis (Zhou et al. 2018). miR-193a/b-3p, which was downregulated in liver tissues of mice with concanavalin A-induced liver fibrosis, has been shown to relieve hepatic fibrosis through suppressing the proliferation and activation of HSCs (Ju et al. 2019). miR-223 is a miRNA involved in the pathogenesis of various liver diseases (Ye et al. 2018) and has been identified as a novel anti-inflammatory and anti-fibrotic therapeutic target (Calvente et al. 2019). Several studies have demonstrated that miR-223-3p alleviated hepatic fibrosis and suppressed HSC activation, confirming the hepatoprotective role of miR-223 (Jimenez Calvente et al. 2020; Liu et al. 2019a). Consistent with the anti-fibrotic effect of miR-223 described above, in this study, we experimentally confirmed that the inhibitory effect of NK-Exo treatment on TGF-β-induced HSC activation was mediated by miR-223 delivery.

A previous study showed that miR-223 inhibited hep3B (hepatocellular carcinoma cell line) cell proliferation and promoted apoptosis by directly targeting NLRP3 (NOD-like receptor family, pyrin domain containing 3) (Wan et al. 2018). Evidence indicates that activation of NLRP3 inflammasome can lead to HSC activation and liver fibrosis (Jiang et al. 2017; Inzaugarat et al. 2019). Therefore, we speculate that miR-223 may also attenuate HSC activation by inhibiting the NLRP3 inflammasome via targeting NLRP3. This speculation, however, awaits further validation.

The specific role of autophagy in HSC activation and liver fibrosis is sometimes contradictory. On one hand, autophagy induction has been shown to promote HSC activation and liver fibrosis. Ye et al. recently reported that ursodeoxycholic acid attenuated TGF-β1-induced HSC activation and alleviated liver fibrosis in a rat model of CCl_4_-induced fibrosis by inhibition of autophagy (Ye et al. 2020). Also, Liu et al. proposed that isorhamnetin suppressed liver fibrosis and HSC activation in mouse models of liver fibrosis by reducing extracellular matrix formation and autophagy (Liu et al. 2019b). Consistently, the present study demonstrated that autophagy activation by rapamycin impaired the inhibitory effect of NK-Exo on TGF-β-induced HSC activation. Our findings manifested that autophagy is a pro-fibrogenesis mechanism and autophagy inhibition may alleviate HSC activation and liver fibrosis. However, on the other hand, autophagy has also been suggested to protect against liver fibrosis. Insufficient autophagy has been implicated in liver fibrosis (Hazari et al. 2020). Ma et al. reported that ampelopsin, a natural flavonoid, attenuated CCl_4_-induced mouse liver fibrosis and HSC activation by inducing autophagy (Ma et al. 2019). Seo et al. found that Src inhibition increased autophagy flux and alleviated liver fibrosis and HSC activation (Seo et al. 2020). The protective effect of autophagy was contradictory to our results. Hence, the interactions between autophagy and HSC activation in the process of liver fibrosis are complicated due to diverse etiology and different types of cell settings, which require further investigation.

ATG7 is an autophagy marker. Yu et al. demonstrated that miR-96-5p prevented HSC activation by inhibiting autophagy via directly targeting ATG7 (Yu et al. 2018). In this study, ATG7 was confirmed as the direct target of miR-223 using the luciferase activity assay. Furthermore, ATG7 overexpression in HSC-LX2 cells abolished the inhibitory effect of NK-Exo on TGF-β1-induced HSC activation. These results suggested that the mechanism by which NK-Exo attenuated TGF-β-induced HSC activation was related to exosomal miR-223 inhibition of autophagy via targeting ATG7.

In summary, the present study demonstrated for the first time that exosomal miR-223 derived from NK cells attenuated TGF-β1-induced HSC activation by suppressing autophagy via targeted inhibition of ATG7. Regulation of miR-223 expression in exosomes released from NK cells might be a putative therapeutic strategy against liver fibrosis.

## Supplementary information

**Additional file 1: Supplementary Figure 1.** Characterization of NK-Exo. **A.** Morphological characterization of NK-Exo by TEM. **B.** The size distribution of NK-Exo was evaluated by the nanoparticle tracking analysis. **C.** The protein levels of the exosomal markers ALIX and CD63 were examined by western blot.

## Data Availability

The datasets used and/or analysed during the current study are available from the corresponding author on reasonable request.

## Abbreviations

3′-UTR: 3′-untranslated region
ATG7: Autophagy-related 7
CCl_4_: Carbon tetrachloride
CoL1A1: Collagen type I alpha 1 chain
HSC: Hepatic stellate cell
miRNA: MicroRNA
Mut: Mutated
NASH: Nonalcoholic steatohepatitis
NC: Negative control
NK: Natural killer
NLRP3: NOD-like receptor family, pyrin domain containing 3
qRT-PCR: Quantitative real-time PCR
TEM: Transmission electron microscopy
WT: Wild-type
α-SMA: α-smooth muscle actin
