# Supplementary figures and images for: Exosomal miR-223 derived from natural killer cells inhibits hepatic stellate cell activation by suppressing autophagy
Source: Mol Med. 2020 Sep 1;26:81. doi: 10.1186/s10020-020-00207-w (PMC7465359; doi:10.1186/s10020-020-00207-w)

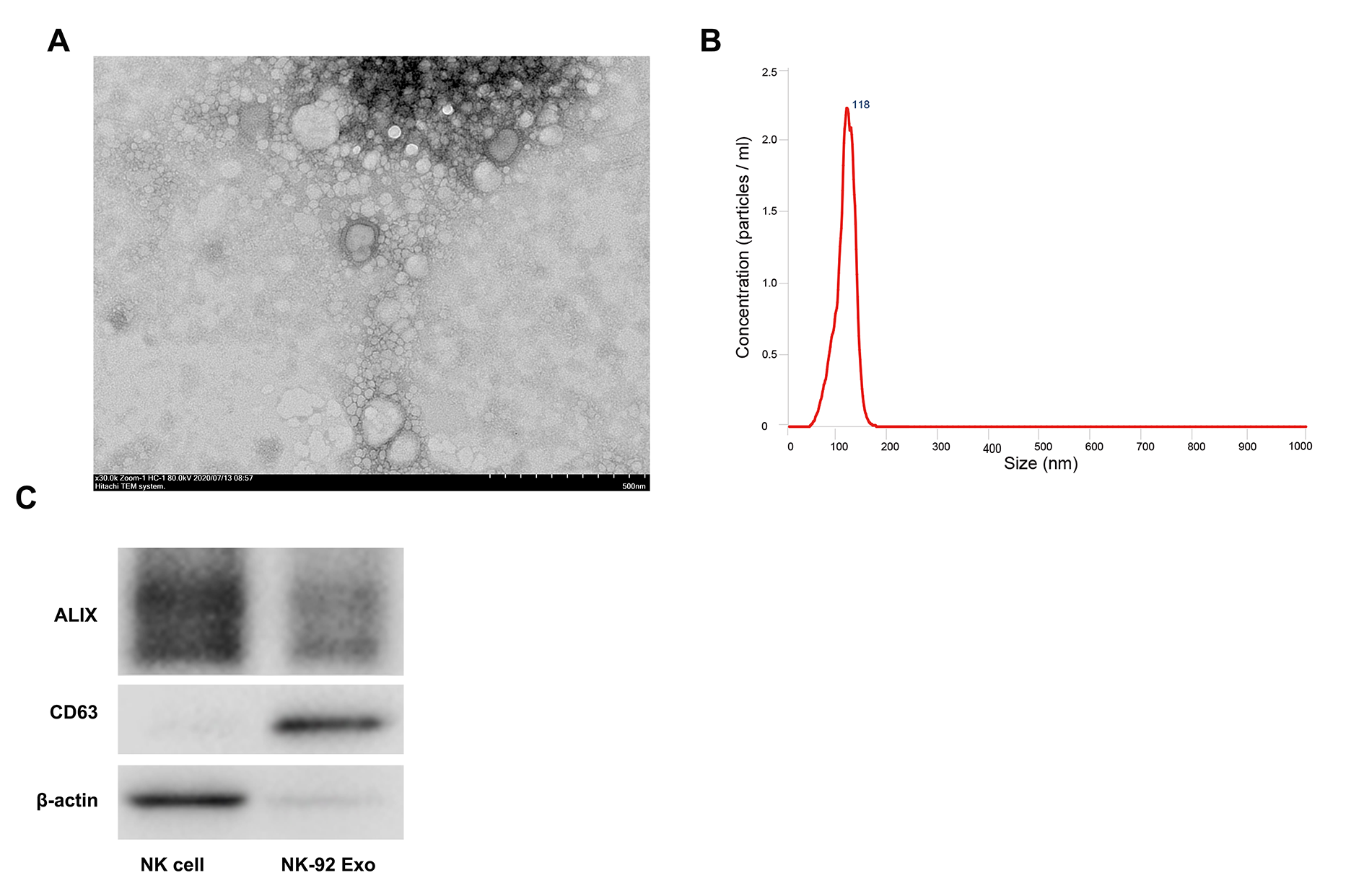

Supplement: Supplementary file 1 — Additional file 1: Supplementary Figure 1. Characterization of NK-Exo. A. Morphological characterization of NK-Exo by TEM. B. The size distribution of NK-Exo was evaluated by the nanoparticle tracking analysis. C. The protein levels of the exosomal markers ALIX and CD63 were examined by western blot. [file 10020_2020_207_MOESM1_ESM.tif]
